# Cultural differences in beliefs and believing about the self – A brain imaging approach

**DOI:** 10.3389/fnbeh.2022.962225

**Published:** 2022-07-22

**Authors:** Shihui Han

**Affiliations:** Beijing Key Laboratory of Behavior and Mental Health, School of Psychological and Cognitive Sciences, PKU-IDG/McGovern Institute for Brain Research, Peking University, Beijing, China

**Keywords:** culture, belief, believe, fMRI, medial prefrontal cortex

## Introduction

Beliefs refer to “fundamental representations of imaginative and emotional content that link an individual’s prior experience with his/her future behavior” (Seitz, 2022). We have beliefs about our societies, other people, and ourselves. When I say “I believe I will be able to contribute an article to this book by working with my colleagues,” I express beliefs about myself and other people that help my decision-making regarding actions in the future. However, due to differences in social information that individuals receive and differences in sociocultural environments in which individuals develop and live, beliefs vary greatly across different human societies. Distinct beliefs not only influence people’s behaviors but also shape how their brains work. In this study, I sought to briefly summarize previous research on cultural differences in beliefs about the self and relevant neural underpinnings. I further introduce a recent brain imaging approach to the neural correlate of believing and its potential cultural differences. Finally, I discuss the implications of the transcultural brain imaging findings related to belief and believing.

## Cultural differences in beliefs

What is the nature of the self as a social unit in human societies? Representations of the self are built based on individuals’ prior experiences and guide their social/economic/political decision-making. Understanding beliefs about the self has been the goal of research in multiple disciplines including philosophy, psychology, and neuroscience. Interestingly, beliefs regarding the self vary tremendously across different populations and different societies and have distinct neural correlates. For example, Western thoughts regard the self as a particular being that is distinct from others (Seigel, 2005) whereas Chinese thoughts take the self as a knot in a social network that unifies numerous individuals as a whole (Zhang, 2005).

These philosophical thoughts are formulated in psychology by Markus and Kitayama (1991) who claimed that Western (European and North American in particular) cultures teach people to view the self as an independent and autonomous entity that is inclined to attend to the self-more than others and emphasizes unique dispositions or traits of the self (an independent view of the self). East Asian cultures, however, view the self that is fundamentally connected with others and thus are sensitive to information about significant others. The unique cultural beliefs about the self are tested empirically in both behavioral and brain imaging studies. It has been shown that Westerners remember self-related information better than information about close others such as mother and best friend (Klein et al., 1989; Heatherton et al., 2006), whereas Chinese remember information about self and close others equally well (Zhu and Zhang, 2002). These findings suggest distinct cultural beliefs about the self and are supported by transcultural brain imaging research on neural underpinnings of representations of the self.

An early functional magnetic resonance imaging (fMRI) study scanned both Chinese and Western students during judgments on personality traits of the self and a close other (i.e., mother) (Zhu et al., 2007). The results revealed activations in the ventral medial prefrontal cortex (mPFC) in response to the reflection of one’s own traits in both Chinese and Western students. However, reflections of the self and mother evoked overlapping mPFC activity in Chinese, whereas Westerners showed greater mPFC activity during reflection of the self (vs. mother). These fMRI findings implicate overlapping neural representations of beliefs about the self and a close other in Chinese but not in Western students. A following fMRI study scanned both Chinese and Danish students, using fMRI, while the participants reflected personality traits, physical features, or social roles of themselves or a familiar celebrity in their own country (Ma et al., 2014). It was found that, although both Chinese and Danish students showed increased ventral mPFC activity during self-reflection of the self (vs. celebrity), the ventral mPFC activity was greater in Danish than in Chinese students. However, self-reflection on social roles in Chinese but not Danish students activated the temporoparietal junction, which is engaged in the processing of others’ minds (Saxe and Kanwisher, 2003). In addition, Chinese compared to Danish students showed stronger functional connectivity between the ventral mPFC and the TPJ associated with self-reflection on social attributes.

Beliefs about the self also differ substantially between religious believers and non-believers. An fMRI study of non-religious and Christian Chinese found that, while the non-religious participants showed activations in the ventral mPFC during reflection of the self, self-reflection activated the dorsal mPFC in Christians (Han et al., 2008). Because the dorsal mPFC is usually involved in the inference of others’ mental states (Grèzes et al., 2004), it was speculated that the dorsal mPFC supports beliefs regarding the self that underscores the evaluative process of the self by God. An fMRI study of Chinese Buddhists showed that reflection of the self-activated the dorsal mPFC, the rostral anterior cingulate and midcingulate cortices, and the left frontal/insular cortices (Han et al., 2010). It is likely that, during the self-reflection task, Buddhists might have to monitor the conflict between the doctrine of no-self and self-focus thinking during self-trait judgment. Together, these brain imaging findings suggest that, in response to cultural group differences in beliefs regarding the self, the human brain has evolved distinct patterns of neural activities that support specific beliefs about the self.

## Cultural differences in believing

Early brain studies focused on cultural differences in neural representations of beliefs regarding the self. Little research examined neural correlates of believing as a process. Conceptually, believing may include multiple mental operations that support perception, valuation, information storage, and prediction (Angel and Seitz, 2016). Methodologically, it is a challenge to disentangle the neurocognitive processes of believing from other mental processes by controlling perceptual/cognitive/affective processes that do not essentially characterize believing. Based on the assumption that the believing process is connected with personal relevance and deals with a set of knowledge with a hierarchically organized structure (Sugiura et al., 2015), we designed a believing task and a control task in an fMRI study to disentangle neural processes of believing (Han et al., 2017). “Believe” and “think” are two words that are used to mutually explain each other in lay opinions (Allen et al., 1990) and may consist of overlapping mental processes. While being presented with trait adjectives during fMRI scanning, one group of Chinese participants was asked to make a yes or no response to the question “Do you *believe* that the trait adjective describes you (or a celebrity)?” (Belief group). Another group of Chinese participants made a yes or no response to the question “Do you *think* the trait adjective describes you (or a celebrity)?” (Think group). The same set of trait adjectives was used in different judgment tasks and judgments on a celebrity were employed as a control condition. We examined believing specific neurocognitive processes by comparing brain activities of Believe vs. Think groups, which controlled control irrelevant perceptual, memory, and semantic processes of stimuli and motor responses. We found that the believing tasks relative to thinking tasks resulted in better memory of self-related adjectives. In addition, believing compared to thinking tasks were associated with stronger activations in the left anterior insula/inferior frontal cortex and stronger functional connectivity between the mPFC and the left occipital cortex. These results provide preliminary evidence for distinct neurocognitive processes involved in believing.

Gao et al. (2022) further tested possible cultural differences in neurocognitive processes underlying believing by collecting behavioral and fMRI responses from a Chinese and a Danish sample in the believing task used in Han et al. (2017). Reaction times and response types (yes or no responses) during believing judgments on the self and a celebrity were collected from the two cultural groups and subject to the drift-diffusion model (DDM) analyses. The results revealed three differences in cognitive processes that characterize the believing task between Chinese and Danes. First, positive and negative trait adjectives shifted the posterior distributions of the drift rate in DDM (as an index of the speed of information acquisition) either lower or larger than zero during both self- and celebrity-believing in Chinese but not in Danes. These results suggest that information acquisition during believing tasks was more sensitive to emotional contexts produced by semantic meanings of trait adjectives in Chinese. Second, the analyses of the non-decision time in DDM (as an index of processes irrelevant to decision-making) showed evidence for overlapping non-decision processes involved in self- and celebrity-believing judgments in Chinese but not in Danes. Third, the analyses of the threshold separation in DDM (as an index of decision-making strategy) suggest that the Chinese were more cautious during celebrity- than self-believing judgments whereas Danes were more cautious during self- than celebrity-believing judgments. These behavioral results are consistent with previous findings that uncovered context-dependent processing in East Asians but context-independent processing in Westerners in multiple levels of cognitive processes (e.g., Kuühnen and Oyserman, 2002; Han et al., 2011) and overlapping processes of the self and significant others in East Asians but not in Westerners (Markus and Kitayama, 1991; Zhu et al., 2007).

fMRI results of Gao et al. (2022) also revealed evidence for distinct neural activities involved in believing in the two cultural groups. Believing judgments activated the mPFC in both Chinese and Danes. However, believing judgments elicited stronger activities in the left anterior insular and ventral frontal activations in Chinese compared to Danes. In addition, Chinese participants with greater mPFC activity showed a longer duration of non-decision processes during believing-judgments. By contrast, greater mPFC activity predicted a lower degree of adopting a conservative strategy during believing judgments in Danes.

Together, the findings of cultural group differences in behavioral and neural responses during the believing task suggest that believing may be decomposed into separate processes such as information acquisition, non-decision processes, and response strategies (e.g., degree of cautiousness) that, respectively, undergo influences of individuals’ cultural experiences. In addition, our results may be interpreted as that the believing task may engage deeper processing of semantic and social knowledge about others in the left ventral frontal cortex in Chinese than in Danes. Even the same brain region (e.g., mPFC) that was observed to be activated during believing in both cultural groups may be linked to different processes of believing (e.g., durations or strategies of the non-decision processes).

## Discussion

The brain imaging findings summarized above have several implications for our understanding of beliefs and believing. First, people from different societies have different beliefs (e.g., different religious beliefs or different beliefs regarding the self). This cultural difference, from a neuroscience perspective, implicates that neural representations of imaginative and emotional content link an individual’s prior experience with his/her future behavior vary across cultures, having in mind that this is critical for cross-cultural communications and social interactions. Second, even though there are correspondent words of “belief” and “believe” in different languages, an apparently same belief (e.g., the belief regarding the self) may have different meanings for people in different cultural environments. This implies that cognitive and neural representations of a belief may be discrepant in people from different cultures. Third, neural processes engaged in believing may also vary greatly in people from different cultures, which may reflect the consequence of social learning of beliefs that have distinct social motivations and goals in different societies.

Finally, cultural differences in beliefs are not a purely mental phenomenon but have biological underpinnings. Development of culturally specific beliefs and belief processes may be understood from the perspective of the culture–behavior–brain loop model of human development (Han and Ma, 2015; Han, 2017), which suggests both indirect culture–brain interactions, through the practice of behaviors, and direct culture–brain interactions, which constitute an interacting loop that provides a basis of human development. Shared beliefs provide a bridge to link social behavior and the brain and guide their interactions in a specific socio-cultural environment, which in turn results in the development of distinct neurocognitive processes underlying belief. Finally, it should be acknowledged that the current studies of beliefs and beliefs focused on a specific topic (e.g., self) and tested small samples of limited cultural groups. Future research may expand this line of research to other beliefs by designing new believing tasks and collecting behavioral and brain imaging data from other large cultural samples.

## Author contributions

The author confirms being the sole contributor to this work and has approved it for publication.

## Conflict of interest

The author declares that the research was conducted in the absence of any commercial or financial relationships that could be construed as a potential conflict of interest.

## Publisher’s note

All claims expressed in this article are solely those of the authors and do not necessarily represent those of their affiliated organizations, or those of the publisher, the editors and the reviewers. Any product that may be evaluated in this article, or claim that may be made by its manufacturer, is not guaranteed or endorsed by the publisher.
